# Estimation of Supercapacitor Energy Storage Based on Fractional Differential Equations

**DOI:** 10.1186/s11671-017-2396-y

**Published:** 2017-12-22

**Authors:** Ryszard Kopka

**Affiliations:** Opole University of Technology, ul. Prószkowska 76, Opole, 45-758 Poland

**Keywords:** Supercapacitor, Supercapacitor energy storage, Fractional differential equations, Parameter estimation

## Abstract

In this paper, new results on using only voltage measurements on supercapacitor terminals for estimation of accumulated energy are presented. For this purpose, a study based on application of fractional–order models of supercapacitor charging/discharging circuits is undertaken. Parameter estimates of the models are then used to assess the amount of the energy accumulated in supercapacitor. The obtained results are compared with energy determined experimentally by measuring voltage and current on supercapacitor terminals. All the tests are repeated for various input signal shapes and parameters. Very high consistency between estimated and experimental results fully confirm suitability of the proposed approach and thus applicability of the fractional calculus to modelling of supercapacitor energy storage.

## Background

As of today, supercapacitors are the main components of many devices and systems, e.g., backup power and electricity recovery systems as well as automotive applications, hybrid vehicles and many others. The ability to accumulate charge without any chemical reactions makes such elements to have hundreds of times higher number of charge/discharge cycles in comparison to typical batteries [1]. Additionally, high charge/discharge rates make them effective for applications in energy recovery systems used for example in transportation or renewable energy sources [2, 3]. In all these applications, the key parameter is the information on the amount of accumulated energy in the supercapacitor [4, 5]. Unfortunately, the well known relationship for typical capacitors that allows to determine the information, that is (1/2)*CU*^2^, cannot be used [6]. The amount of accumulated energy cannot be determined on the basis of the voltage on capacitor terminals only. The main reason for this is the diffusion process associated with the charge redistribution [1, 7]. This is why many researchers have been trying to determine a supercapacitor model that would allow estimating the behavior of a real system. Currently, researchers mainly adopt the combinations of typical electronic elements, e.g., *RC* quadripole or series and parallel combinations of such elements. However, all of these models assume a relationship between supercapacitor current and voltage on its terminal in form of a typical, integer order differential equation [3–5, 7].

But it turns out that some completely new possibilities for energy estimation in such systems can be obtained by the application of the fractional calculus [8, 9]. The noninteger–order differo–integral calculus was proposed over 300 years ago, but important implementation issues are related with the advent of computers and their use in modeling of discrete–time dynamical systems [10–14]. Application of fractional calculus to the problem of supercapacitor parameter estimation is not a new issue. There are many publications in this field [15–25]. The authors perform the task of estimating parameters in both frequency and time domains [26].

This paper is an extended version of the author’s conference presentation [27], in which a fractional–order approach has been briefly introduced to estimate energy accumulated in the supercapacitor.

Accurate estimation of parameters of the supercapacitors is also of utmost importance in assessing their reliability [28–31]. Permanent degradation processes inside the supercapacitor can change the equivalent series resistance and capacitance. Thus, accurate determination of these parameters, based on the proposed method, also allows to accurately assess the performance of the capacitor.

This paper starts with some preliminaries related to fractional–order integration and differentiation. Next, it presents the parameter estimation method used during tests and proposes new energy calculation method based on the fractional calculus. The Results and Discussion section present the calculated energy for various scenarios and compare them with reference (measured) values. Conclusions and contributions are summarized in Conclusions section.

## Methods

The use of porous materials in supercapacitors and specific manner of charge accumulation cause that the traditional approaches based on integer order derivative models are not accurate enough. Many researchers have proposed various solutions in form of combination of typical *RC* elements with constant or variable values [4, 7]. But it turns out that definitely better precision can be obtained using noninteger–order differential calculus for defining the relationships between supercapacitor’s current and voltage [17, 19]. Additionally, such a solution may result in a very simple model structure, while providing very high accuracy [18].

### Fractional Order Differo–Integral Calculus

Fractional order differential calculus has been known for over 300 years. However, only recent several years have brought its popularity in modelling of physical phenomena and processes. It is believed that description of dynamics with a derivative or integral of noninteger–order can be one of the most effective methods for modelling of real properties of many complex phenomena and industrial processes, especially based on novel materials and technologies [10, 12, 13, 32–34].

Noninteger–order differential or integral calculus is a generalization of classical calculus to order *α* that belongs to the set of real numbers $\mathcal {R}$. The differo–integral operator of order $\alpha \in \mathcal {R}$ of function *f*(*t*) on the range [*a*,*t*] can be written as follows

1 $$ {{}_{a}\mathcal{D}_{\textit t}^{\alpha}}f(t)= \left\{ {\begin{array}{l c l} {\frac{\mathrm{d}^{\alpha}\textit{f(t)}}{\mathrm{d} \textit{t}^{\alpha}}} & \text{for} & \alpha>0\\ f(t) & \text{for} & \alpha=0\\ \int_{a}^{t} f(\tau)\textrm {d} {\tau^{\alpha}} & \textrm {for} & \alpha<0,\\ \end{array}} \right.  $$

assuming that the function *f*(*t*) is multiple times differentiable and integrable. As for the operator (1), there are many definitions of its realization. Such definitions differ in properties and areas of application. The most popular are the Riemann–Liouville, Caputo and Grünwald–Letnikov (GL) definitions [34]. The latter will be used in this paper in the form

2 $$ {}_{a}\mathcal{D}_{t}^{\alpha} f(t) = {\lim}_{h \to 0} \frac{1}{h^{\alpha}} \sum\limits_{j=0}^{\left[{\frac{t}{h}}\right]}(-1)^{j}{\alpha \choose j}f(t-jh),  $$

where the binomial $\alpha \choose j$ is defined as follows

3 $$ {\alpha \choose j}= \left\{ \begin{array}{lll} 1 & \textup {for} &j=0 \\ \frac{\alpha (\alpha-1) \dots (\alpha-j+1)}{j!} & \text{for} & j>0. \end{array} \right.  $$

In order to obtain a fractional model at discrete moments of time, the GL definition in a discrete form is simplified as

4 $$ \Delta_{h}^{\alpha} f(t) = \frac {1}{h^{\alpha}} \sum\limits_{j=0}^{t}(-1)^{j}{\alpha \choose j}f(t-j).  $$

There are several discretization schemes for the GL Eq. (4). The most popular ones include backward differences (Euler), trapezoidal (Tustin), and Al Alaoui operators. Using Euler’s method, the fractional derivative at discrete time moments *k* can be presented as

5 $$ \Delta_{h}^{\alpha} f(k)=\frac {1}{h^{\alpha}}\sum\limits_{j=0}^{k}(-1)^{j}{\alpha \choose j}f(k-j),\; k=0,1,\ldots.  $$

The infinite sum of previous samples must be in real systems limited to a finite value due to the limited memory and limited calculation time. Now, the truncated or finite–length discrete–time approximation of GL is

6 $$ \Delta^{\alpha} f(k) =\frac {1}{h^{\alpha}}\sum\limits_{j=0}^{L}(-1)^{j}{\alpha \choose j}f(k-j),\; k=0,1,\ldots,  $$

where *f*(*l*)=0 for *l*<0 and *L* is the length of the model (6) [23]. Reducing the number of samples results in decreased calculation accuracy. This is important for systems operating in a continuous time. Some other kinds of solution are algorithms approximating fractional differo–integrals with integer order models. An example can be Oustaloup recursive filters [35]. Another effective finite–length model is the FFLD, being a combination of the truncated model (6) and a Laguarre–based difference [24, 36, 37].

**Fig. 1:**
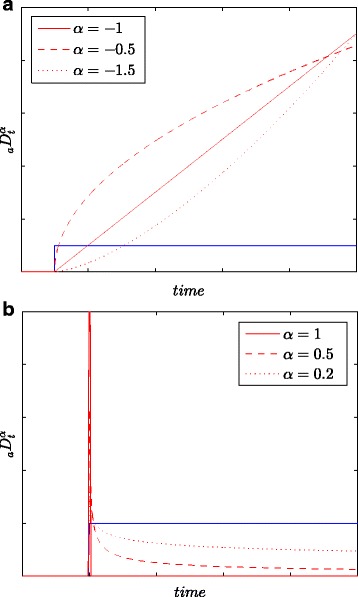
Step responses for integrating (**a**) and differentiating (**b**) models with various orders *α*

All results of identification as well as energy measurements are obtained based on all samples in the (long) observation window *L*, i.e., with maximum accuracy. Figure 1 presents the step responses of integration and differentiation obtained based on (6), for *k*=0,1,…,*L* and for various values of integration/differentiation order *α*. Assuming different values of order *α*, one can more accurately model different physical processes, especially diffusion ones.

### Parameter Estimation for Fractional Model

**Fig. 2:**
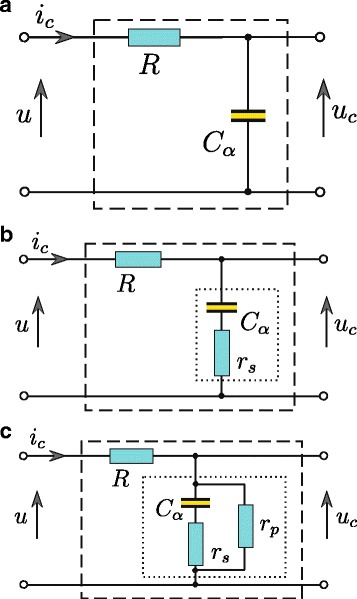
Supercapacitor *RC* models, base model (**a**), expanded with a series resistance (**b**), and with additional parallel resistance (**c**)

The results of all energy measurements and identification procedures presented in this paper were obtained for a supercapacitor charged from a controlled voltage source. In such a system, the supercapacitor current *i*_*C*_(*t*) must be limited by a resistor *R* connected in series with the supercapacitor *C* (Fig. 2). Estimation of all supercapacitor parameters is performed based on quadripole response *u*_*C*_(*t*) to voltage step *u*(*t*) at its input. Choosing the appropriate value of derivative order *α* allows to account for a supercapacitor model of the physical phenomena related to diffusion processes associated with the charge redistribution during the charging and discharging processes. The parallel resistor *r*_*P*_ additionally enables modelling of the leakage current. Using the fractional differential calculus for modeling supercapacitors, the model structure can be of low complexity. For supercapacitor charged from the voltage source, a model consists of only two elements, i.e. a simple *RC* quadripole (Fig. 2a). For low capacities, the series resistance *r*_*S*_ is of importance (Fig. 2b), while the leakage current *I*_*L*_ may be additionally represented by the parallel resistance *r*_*P*_ (Fig. 2c). Using the fractional order calculus to model the supercapacitor, the relation between the voltage on capacitor terminals and its current can be expressed as follows

7 $$ i_{C}(t)=C_{\alpha}\frac{\mathrm{d}^{\alpha} u_{C}(t)}{\mathrm{d} t^{\alpha}},  $$

where the operator d^*α*^/d*t*^*α*^ means a differentiation operator of order *α* and the SI unit of *C*_*α*_ is [F/sec^1−*α*^]. The basic supercapacitor configuration presented in Fig. 2a can be treated as a first–order inertial system and can be represented by the fractional transfer function

8 $$ G(s^{\alpha})=\frac{U_{C}(s)}{U(s)}=\frac{1}{Ts^{\alpha} +1},  $$

where *T*=*RC*_*α*_. Taking into account the series resistance *r*_*S*_ (Fig. 2b), the circuit is treated as phase–delaying correction system with the transfer function (compare [24])

9 $$ G(s^{\alpha})=\frac{1}{T_{1}s^{\alpha}+1}+\frac{T_{2}s^{\alpha}}{T_{1}s^{\alpha}+1},  $$

where *T*_1_=*C*_*α*_(*R*+*r*_*S*_) and *T*_2_=*r*_*S*_*C*_*α*_. Additionally, allowing for the parallel resistor *r*_*P*_ representing the leakage current *I*_*L*_ (Fig. 2c), the system transfer function can be expressed as

10 $$ G(s^{\alpha})=\frac{T_{2}s^{\alpha} +1}{T_{1}s^{\alpha} +K},  $$

where *K*=*R*/*r*_*P*_+1, *T*_1_=*C*(*Rr*_*s*_/*r*_*P*_+*R*+*r*_*S*_) and *T*_2_=*r*_*S*_*C*. In the time domain, Eq. (10) can be presented as

11 $$ \frac{\mathrm{d}^{\alpha} u_{C}(t)}{\mathrm{d}t^{\alpha}}=\frac{1}{T_{1}}(u(t)-Ku_{C}(t))+\frac{T_{2}}{T_{1}}\frac{\mathrm{d}^{\alpha} u(t)}{\mathrm{d}t^{\alpha}}.  $$

**Fig. 3:**
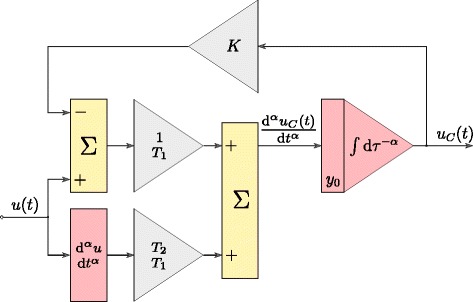
Matlab structure of supercapacitor model in time–domain

**Fig. 4:**
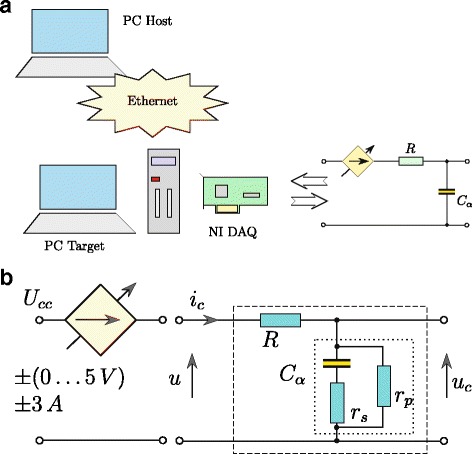
Structure of measurement system (**a**) and supercapacitor charging/discharging scheme (**b**)

The time response of the model defined by (11) was obtained by transforming it into the form presented graphically in Fig. 3, where integration and differentiation operations are of fractional order *α*. This model was used during the process of estimation of supercapacitor parameters. The tested supercapacitor was identified using the system presented in Fig. 4a. The control procedure of the entire system was developed using the Matlab/Simulink software with xPC Toolbox. The system consisted of a desktop PC (xPC Target) with the installed measurement card NI-DAQ and master computer (xPC Host). The computers were interconnected through the Ethernet network. The supercapacitor was charged and discharged by (voltage–controlled) voltage source (Fig. 4b) of current efficiency up to ± 3 A. The measurement system was operated with the sampling frequency of 100 Hz, while all the measurements and analog control signals were processed with 16–bit resolution [25].

**Fig. 5:**
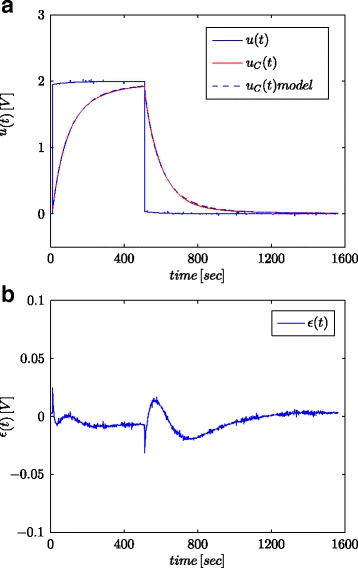
Step responses for tested supercapacitor and its fractional model (**a**) and the model response error (**b**)

**Fig. 6:**
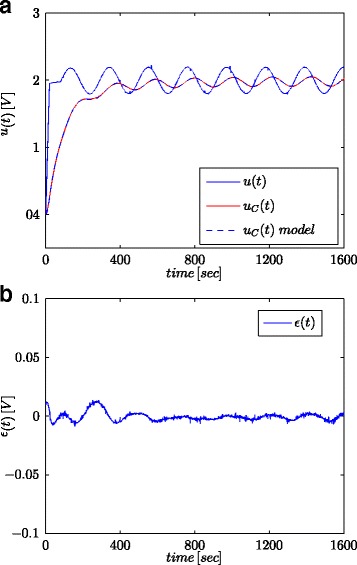
Sinusoidal wave responses for tested supercapacitor and its fractional model (**a**) and the model response error (**b**)

The main method for determining the dynamic properties of a system is based on the analysis of the step response [38]. In relation to the system model, this method allows estimation of its parameters. For this study, the step signal with various voltages (0.5/1.0/1.5/2.0/2.7 V) and constant duration (500 s) have been used (see Fig. 5 and Table 2). On the other hand, one of the typical applications of supercapacitors is the accumulation or delivery of energy into the power systems. In this case, the voltage change rate is rather small. To simulate it, the 400 mVpp and 0.03 rad/s signal with 2 V offset was used (Fig. 6). Additionally in order to examine the influence of the voltage and frequency changes on estimated parameters, various values of the latter were used (see Table 3).

**Table 1:** Results of identification of *RC* quadripole for nominal parameters: *C*_*n*_=100 *F*, *U*_*n*_=2.7 *V* and *R*=0.995 *Ω*

Parameter		Step response	Sinusoidal response
*C*_*α*_	*F* *s* ^*α*−1^	63.80	64.38
*r*_*S*_	*m* *Ω*	3.80	0.86
*r*_*P*_	*Ω*	2.19×10^49^	3.86×10^36^
*α*	−	0.9157	0.9196

*Note:* *C*_*α*_ – fractional capacitance

**Table 2:** Estimates of parameter vector *θ*=[*α*,*C*_*α*_,*r*_*S*_,*r*_*P*_] for various voltage steps excitations, *C*_*n*_=100 *F*, *U*_*n*_=2.7 *V* and *R*=0.985 *Ω*)

*U*_*step*_	*α*	*C*_*α*_	*r*_*S*_	*r*_*P*_
[*V*]	–	*F* *s* ^*α*−1^	*m* *Ω*	*Ω*
0.5	-0.9594	84.9	17.7	1.28e+16
1.0	-0.9561	84.9	37.4	1.19e+21
1.5	-0.9398	79.5	16.1	1.46e+16
2.0	-0.9006	68.4	12.9	6.20e+17
2.7	-0.8788	63.8	9.4	1.45e+14

**Table 3:** Estimates of parameter vector *θ*=[*α*,*C*_*α*_,*r*_*S*_,*r*_*P*_] for sinusoidal excitation with various frequencies and amplitudes, *C*_*n*_=100 *F*, *U*_*n*_=2.7 *V* and *R*=0.985 *Ω*, *U*_*offset*_=2.0 *V*)

		Frequency			
			0.01 rad/s	0.03 rad/s	0.05 rad/s
Amplitude	0.10 V	*α*/−	-0.9634	-0.8870	-0.9616
		*C*_*α*_/*Fs*^*α*−1^	81.7	64.9	82.7
		*r*_*S*_/*mΩ*	34.3	2.2	18.6
		*r*_*P*_/*Ω*	4.6e+18	4.4e+21	6.6e+17
	0.25 V	*α*/−	-0.9501	-0.9287	-0.9524
		*C*_*α*_/*Fs*^*α*−1^	76.8	74.1	78.6
		*r*_*S*_/*mΩ*	110	22.6	27.8
		*r*_*P*_/*Ω*	7.4e+14	1.9e+19	3.7e+19
	0.50 V	*α*/−	-0.9476	-0.9433	-0.9568
		*C*_*α*_/*Fs*^*α*−1^	75.8	75.2	78.3
		*r*_*S*_/*mΩ*	46.8	17.5	26.3
		*r*_*P*_/*Ω*	4.1e+18	1.1e+20	1.1e+19
	0.70 V	*α*/−	-0.8938	-0.8972	-0.9566
		*C*_*α*_/*Fs*^*α*−1^	63.12	64.17	78.7
		*r*_*S*_/*mΩ*	23.8	3.3	23.1
		*r*_*P*_/*Ω*	1.7e+18	1.4e+22	2.1e+24

There are several methods for estimation of model parameters. The main aim of the identification procedure in the time domain applied in this work was to estimate the vector of unknown parameters *θ*=[*α*,*C*_*α*_,*r*_*S*_,*r*_*P*_] of fractional model presented by (11). The least squares method was used to minimize the initial error. An optimization criterion involved minimization of the standard error $\|\epsilon (k)\|_{2}^{2}$, where

12 $$ \epsilon(k)=u_{C}(k)-\hat{u}_{C}(k),  $$

where *u*_*C*_(*k*) is the output voltage measured from the tested system at moment *k*, while $\hat {u}_{C}(k)$ is the output voltage from the considered model for the input signal *u*(*k*). The identification problem is now reduced to finding a parameter vector *θ*∈*Θ*_*ad*_ that would minimize the square criterion *J* in such a manner that

13 $$ \min_{\theta\in\Theta_{ad}} \left\{ J= \sum_{0}^{N} {\epsilon(k)^{T}\epsilon(k)}\right\},  $$

where *Θ*_*ad*_ denotes the set of admissible parameter values and *N* means the simulation time. There are many optimisation algorithms that can be used to solve the problem (13). The results presented in this paper were obtained by implementing the genetic algorithm in the Matlab environment.

### Energy Calculation

A change in the energy stored in the supercapacitor depends on the power supplied to the capacitor per unit of time and can be described as follows

14 $$ \mathrm{d}E(t) =P(t)\mathrm{d}t.  $$

By expressing the power supplied to the capacitor as a product of the current and voltage on capacitor terminals, the change in energy at given time *t* can be expressed as

15 $$ \mathrm{d}E(t) =u_{C}(t)i_{C}(t)\mathrm{d}t.  $$

The total energy during the time interval [*t*_1_,*t*_2_] can be obtained by integrating the energy changes over that time

16 $$ E_{tot}=\int_{t_{1}}^{t_{2}}\mathrm{d}E(t)=\int_{t_{1}}^{t_{2}}u_{C}(t)i_{C}(t)\mathrm{d}t.  $$

Accounting for Eq. (7), the total energy storage can be determined as

17 $$ E_{tot}=C_{\alpha}\int_{t_{1}}^{t_{2}}u_{C}(t)\frac{\mathrm{d}^{\alpha} u_{C}(t)}{\mathrm{d}t^{\alpha}}\mathrm{d}t.  $$

Assuming *t*_1_=0 and $E_{t_{1}}=0$, the total energy stored in the supercapacitor during the time interval [0,*t*] is

18 $$ E(t)=C_{\alpha}\int_{0}^{t}u_{C}(\tau)\frac{\mathrm{d}^{\alpha} u_{C}(\tau)}{\mathrm{d}\tau^{\alpha}}\mathrm{d}\tau.  $$

Note that for *α*=1 Eq. (18) can be reduced to the classical one

19 $$ E(t)=\frac{1}{2}Cu_{C}(t)^{2}.  $$

## Results and Discussion

Initially, the procedure for estimating the parameter vector of the supercapacitor model using the fractional calculus was performed. The estimation was performed based on the system presented in Fig. 2c, generating a voltage step or sinusoidal wave at its input. The model responses were calculated based on (11). The results obtained by the two identification procedures are very similar, especially in the case of fractional capacitance *C*_*α*_ and the fractional order *α* (see Table 1). Some differences in the estimates of series resistance *r*_*S*_ may be a result of its dependence on frequency. The step signal consists of many high frequency harmonics while the sinusoidal wave only one – the 0.03 rad/s. The presented results were obtained for the commercial supercapacitor Samwha Green–Cap EDLC(DB), rated as 2.7 V with 100 F nominal capacitance and 8 m*Ω* maximum equivalent series resistance (*r*_*S*_) at 1 kHz.

Figures 5a and 6a show the measured supercapacitor voltage and the calculated model responses, for step and sinusoidal signals, respectively, while Figs. 5b and 6b show the model response error.

All obtained results show high consistency between model responses and real measurements despite the fact that relatively simple models were proposed. Some discrepancies may result from the fact that model parameters should be estimated in the system of supercapacitor charged and discharged using the current source [25]. Also, very high estimates of *r*_*P*_ may suggest that this resistance could be excluded from the supercapacitor model shown in Fig. 2c. Those very high estimates and their high discrepancies for different inputs indicate that the test signals used to estimate this parameter are not proper. The model (10) was used as the most general form. However, in order to accurately determine all its parameters, it was necessary to use other procedures and test signals. The value of *r*_*P*_ characterizes the leakage current *I*_*L*_ and should be determined using the constant voltage signal, but for a very long time – of order of several dozen hours.

Although the main goal of the study was to measure energy, various excitation conditions largely affected all the parameter estimates (see Table 2). For instance, the increase of the voltage step amplitude significantly changed the fractional integration order, as the result of increasing effect of the diffusion phenomena inside the supercapacitor. It can also be seen from Table 2 that the supercapacitor is quite nonlinear. As a result of the integration order changes, the variation of the fractional capacity is also observed. This also applies to sinusoidal excitation. The values of estimated parameters—especially *α* and *C*_*α*_—depend on the amplitude and frequency (see Table 3). For low frequencies, the amplitude value is important, while for higher frequencies the supercapacitor behaves like being excited with a constant voltage.

### Energy Calculation

**Fig. 7:**
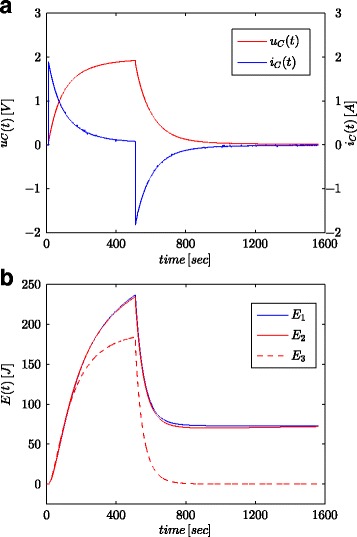
Step responses for supercapacitor voltage and current (**a**) and calculated energy values (**b**)

**Fig. 8:**
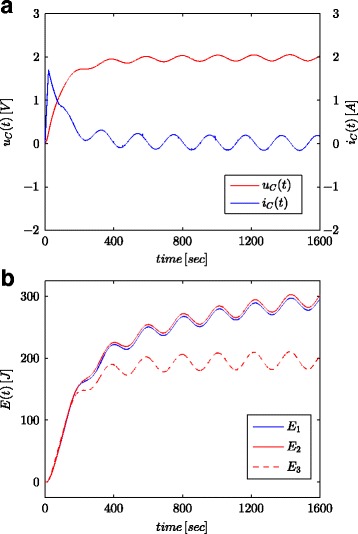
Sinusoidal responses for supercapacitor voltage and current (**a**) and calculated energy values (**b**)

**Fig. 9:**
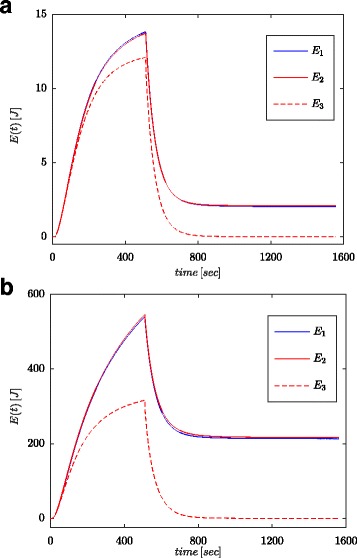
Energy amounts calculated for step excitations of 0.5 V (**a**) and 2.7 V (**b**)

**Fig. 10:**
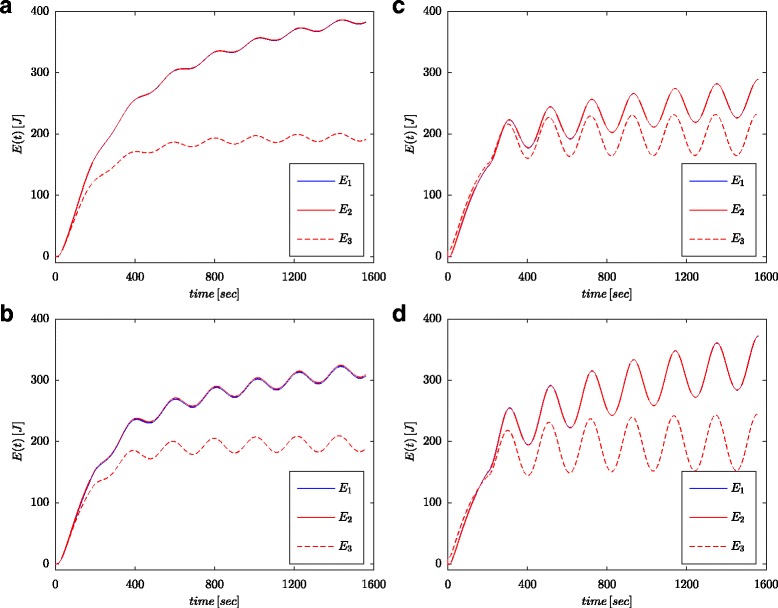
Energy amounts calculated for sinusoidal excitations with frequency 0.03 rad/s and amplitudes 0.1 V (**a**), 0.25 V (**b**), 0.5 V (**c**), and 0.7 V (**d**)

Figures 7a and 8a show measured values of the voltage and current of the supercapacitor for the configuration as presented in Fig. 4b. These values were used for calculation of the total energy stored in the capacitor (marked as *E*_1_ in Figs. 7b and 8b) according to (16). Just as for parameter identification processes, the calculations were conducted both for voltage step and sinusoidal wave at the system input. The energy calculated in such a manner for each time *t* was compared with energy calculated based on the voltage and capacity in accordance with (19) (marked as *E*_3_ in Figs. 7b and 8b) and energy calculated with fractional–order calculus (marked as *E*_2_ in Figs. 7b and 8b) according to (18). For Eq. (19), a nominal value of supercapacitor was adopted (*C*_*n*_), while in (18) the value obtained from the estimation process presented in Table 1 was used. Figure 7b shows results of measurements and energy calculations for voltage step, while Fig. 8b shows this same quantities for sinusoidal wave. Similar calculations were made for different voltage steps and sinusoidal excitations. Figure 9a, b shows an example of measured and calculated energies for two voltage steps of 0.5 V and 2.7 V, respectively. Figure 10 shows the energy changes for a sinusoidal signal with the frequency of 0.03 rad/sec and different amplitudes of 0.1/0.25/0.5 and 0.7 V. It can be seen that the differences in the determined energy values correspond to differences in the estimated values of the fractional order *α*. The greater the difference from the value − 1, the greater is the difference in the calculated energies.

### Discussion

The use of a porous material electrodes in supercapacitors in form of active carbon isolated by a very thin separator and use of charge accumulation mechanisms as so–called double layer, gives an enormous increase in their capacity. However, application of new materials and new design solutions result in the fact that traditional mathematical calculations in form of integer–order derivatives and integrals appear inaccurate. The conducted measurements and calculations prove the fractional–order nature of supercapacitors. By correct estimation of noninteger order *α* of derivative/integral, one can precisely model phenomena and processes occurring inside the supercapacitor using simple mathematical models.

Taking into account the real value of the accumulated energy determined by (16), the integer–order model with nominal parameters (19) under–estimates the amount of energy, while the fractional model (18) indicates almost the same value.

The performed tests and measurements were related to charging and discharging of the supercapacitor by a voltage source. Under industrial conditions, supercapacitors are usually charged and discharged by current sources. This can change the nature of the system because the capacitor is no longer an inertial system but becomes a typical integrating one. However, the measurements conducted by author also indicate the occurrence of diffusion processes in such cases. Anyway, usefulness of the Gründwald–Letnikov derivative/integral is confirmed here. Another issue is related to the implementation of the GL differo–integral operator as, e.g., the finite or truncated GL difference (6), which may be computationally burdensome. In future research, we will compare the Oustaloup [35] and FFLD [24, 36, 37] approximators to effectively solve the implementation issue.

The amount of energy storage in supercapacitor calculated only on the measured value of supercapacitor terminal voltage and using model (19) is not appropriate. The model (19) is only valid if the capacitor current is characterized by the integer order derivative of the capacitor voltage (*i*_*C*_(*t*)=d*u*_*C*_(*t*)/d*t*). This is not true for supercapacitor as a consequence of its construction and used special materials. However, the same problem occurs with very large supercapacitors charged by current source. There are also quite new element as super-batteries. In all these applications, the current changes are not characterized by the integer-order derivative of the terminal voltage as a consequence of the specific properties of these elements.

## Conclusions

In this paper, a new approach to estimation of an amount of energy accumulated in supercapacitors has been presented. The analysis has been conducted taking advantage of certain unique properties of fractional–order models. It has been shown that application of such sophisticated modeling leads to very accurate results, which can be obtained even though the models themselves are not of high complexity. This is due to a natural ability of noninteger order dynamics to model diffusion processes, just like charge redistribution in supercapacitors. The results of this paper have confirmed the fractional nature of supercapacitors.
